# Copper chloride as a conversion-type positive electrode for rechargeable aluminum batteries

**DOI:** 10.1039/c9ra09158k

**Published:** 2019-12-16

**Authors:** Masanobu Chiku, Takeshi Kunisawa, Eiji Higuchi, Hiroshi Inoue

**Affiliations:** Department of Applied Chemistry, Graduate School of Engineering, Osaka Prefecture University Sakai Osaka 599-8531 Japan chiku@chem.osakafu-u.ac.jp

## Abstract

Copper chloride (CuCl_2_) was investigated for the first time as conversion-type positive electrode material in a rechargeable Al battery. The electrode was reversibly charged and discharged in an electrolyte solution of AlCl_3_, dipropylsulfone, and toluene (1 : 10 : 5 molar ratio). The initial discharge capacity was about 370 mA h (g-CuCl_2_)^−1^ at 0.028C-rate (11 mA (g-CuCl_2_)^−1^), which was almost the same as the theoretical value (399 mA h (g-CuCl_2_)^−1^) and higher than that of insertion-type positive electrode materials as used in the rechargeable Al battery. Moreover, a two-stage discharge plateau voltage was observed at 1.5 V and 0.8 V, which was higher than other conversion type positive electrodes for the aluminum rechargeable battery. The high discharge voltage realized a high energy density of 426 mW h (g-CuCl_2_)^−1^, which is the highest energy density compared with other conversion type positive electrodes. Two different strategies were implemented to increase the lifetime of the cell, namely, increasing the upper cut-off voltage and decreasing the particle size of CuCl_2_. The discharge capacity for the electrode at the second cycle was threefold that for a pristine CuCl_2_ electrode.

## Abstract 2

Copper chloride (CuCl_2_) was investigated for the first time as conversion-type positive electrode material in a rechargeable Al battery.

## Graphical abstract

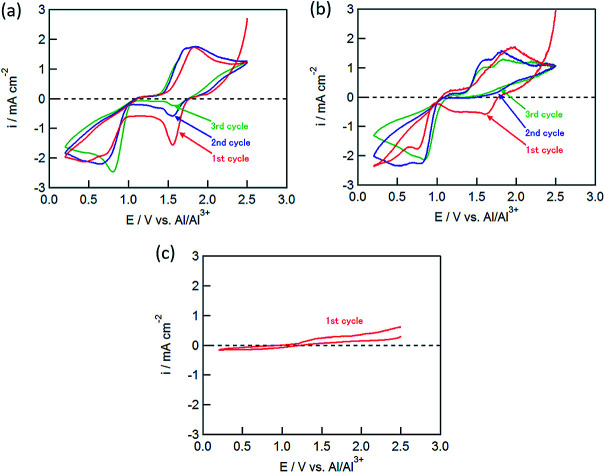

## 1. Introduction

High-performance rechargeable batteries are important components of mobile electronic devices and electric vehicles, and lithium-ion rechargeable batteries (LIBs) are the most common type. The energy densities of LIBs have increased for more than 20 years, and have now reached the theoretical limit. Currently, there is much research on cutting-edge next-generation rechargeable batteries, such as all-solid-state batteries, metal–air batteries, and multivalent cation batteries.^1–4^ Multivalent cations not only have a high energy density but also have higher abundance than Li; moreover, multivalent cation batteries have a similar constitution as conventional secondary batteries which makes it easy to construct them in a factory. Aurbach *et al.* reported the first prototype system for a rechargeable Mg battery.^4^ Mg is a promising negative electrode material for multivalent cation batteries because its volumetric capacity (3830 mA h cm^−3^) is larger than that of Li (2060 mA h cm^−3^); however, Al has an even higher volumetric capacity (8042 mA h cm^−3^), which is about fourfold that of Li.

A new active material for positive electrodes is vital for the development of rechargeable Al batteries. One of the most promising positive electrode materials for multivalent cation batteries is Chevrel-phase Mo_3_S_4_. Aurbach *et al.* first used the Chevrel-phase Mo_3_S_4_ positive electrode in a rechargeable Mg battery; however, the positive electrode potential was low and the operating voltage of the battery was about 1.4–0.8 V.^4^ If the Chevrel-phase Mo_3_S_4_ positive electrode is used in the rechargeable Al battery, the cell voltage will be low. Guo *et al.* reported the Al battery with Mo_3_S_4_ positive electrode, and it show two distinct plateaus at 0.55 and 0.37 V.^5^

Vanadium oxides, which have a layered structure and insert cations between layers for maintaining charge balance, are also promising candidates for positive electrode active materials. So far, V_2_O_5_ nanowires^6^ and hydrothermally deposited V_2_O_5_ (ref. 7) and VO_2_ (ref. 8) have been investigated. We found that amorphous V_2_O_5_ could function as an active material for the positive electrode in a rechargeable Al battery. A maximum discharge capacity of about 200 mA h (g-V_2_O_5_)^−1^ at a rate of 0.025C was obtained.^9^ Amine and co-workers reported that a V_2_O_5_ electrode that was directly deposited onto a Ni current collector exhibited a maximum discharge capacity of 270 mA h (g-V_2_O_5_)^−1^,^10^ which was higher than that of any other oxide based positive electrode active material for the rechargeable Al battery. However, the energy density was not sufficient for commercialization of the device.

To increase the capacity of rechargeable Al batteries, a new approach is necessary.^11–13^ One solution is to devise new positive electrode active materials that have different charge/discharge mechanisms. Transition metal chlorides do not have vacant sites to accommodate Li^+^, but they can function as positive electrode active materials under the following reversible conversion reaction:^14^

1 MCl_*n*_ + *n*Li^+^ + *n*e^−^ ⇄ M + *n*LiCl (M: transition metal)

Transition metal ions are reduced to metal during discharging, and Cl^−^ ions react with Li^+^ ions in the electrolyte to form the ionic compound LiCl.^14^ Thus, dynamic phase changes between MCl_*n*_ and LiCl occur reversibly during the charge/discharge processes, and these processes are different from those for insertion-type positive electrodes. The conversion-type electrode has increased the capacity of LIBs^10^ and may offer a possibility for improving the positive electrode properties of the rechargeable Al battery.

Donahue *et al.* reported the use of FeCl_3_ as the first conversion-type positive electrode active material for the rechargeable Al battery.^15^ Uchimoto *et al.* reported FeS_2_ as conversion type positive electrode material for rechargeable Al battery; however, this battery showed relatively low voltage below 1.2 V and the operation temperature was at 55 °C.^16^ Co/Ni/Cu-based metal-sulfur positive electrodes were also investigated, and Ni_3_S_2_ show 0.8 V plateau voltage and 350 mA h g^−1^ discharge capacity.^17–19^ SnS_2_ was also investigated as conversion type positive electrode material, and the discharge capacity reached 392 mA h g^−1^; however, its discharge voltage plateau was limited as 0.7 V.^20^ In this study, we investigated three transition metal chlorides, namely, CuCl_2_, CuCl, and FeCl_2_, as conversion-type positive electrode materials for the rechargeable Al battery to realize high voltage discharging over 1.5 V at 30 °C.

## 2. Experimental

### 2.1 Materials

Dipropylsulfone (Tokyo Chemical Industry) and all other chemicals (Wako Pure Chemical) were used as received without any purification. Molybdenum plate was purchased from Nilaco Corporation. Ketjen black (KB, EC600JD) was purchased from Lion Corporation.

### 2.2 Preparation of FeCl_2_, CuCl, and CuCl_2_ pellet electrodes

The FeCl_2_, CuCl, and CuCl_2_ powders (88% w/w) were each mixed with KB (10% w/w) and polytetrafluoroethylene (2% w/w) to be 20 mg as total mass, followed by pressing at 290 MPa for 3 min to form pellets 13 mm in diameter with *ca.* 200 μm thickness.

The CuCl_2_ powder was treated by mechanical milling to reduce the particle size. The powder was put into a 45 mL zirconia pot containing seven zirconia balls 10 mm diameter. Mechanical milling was performed with a planetary ball-mill apparatus (Fritsch, Model Pulverisette 7). The milling speed was 157 rpm and the milling times were 0.5, 4, and 12 h.

### 2.3 Electrochemical and spectroscopic characterization

All electrochemical measurements were performed using a lab-built glass cell.^9^ A mixture of aluminum chloride, dipropylsulfone (DPSO_2_), and toluene (1 : 10 : 5 molar ratio) served as the electrolyte solution. A Mo plate (1.3 cm × 1.3 cm), which was used as the current collector, was immersed in conc. HCl for several seconds before use. An Al plate (10 mm in diameter, 0.2 mm in thickness) was used as the counter and reference electrodes in all electrochemical experiments. A glass fiber filter (Advantec, Ltd.) was used as the separator. A test cell was assembled in an Ar-filled glovebox. Cyclic voltammetry and charge/discharge tests were performed at 30 °C using an SI1287 potentiostat (Solartron) and an HJ1001SM8 charge/discharge system (Hokuto Denko), respectively. X-ray diffraction (XRD) spectra were measured by an X-ray diffractometer (50 kV, 30 mA; XRD-6100, Shimadzu) equipped with a Cu Kα source (*λ* = 0.1541 nm). Scanning electron microscope (SEM) images and energy-dispersive X-ray analysis (EDX) were performed with an S-4500 field-emission SEM (Hitachi) and EDAX system (Ametec), respectively.

## 3. Results and discussion

**Fig. 1:**
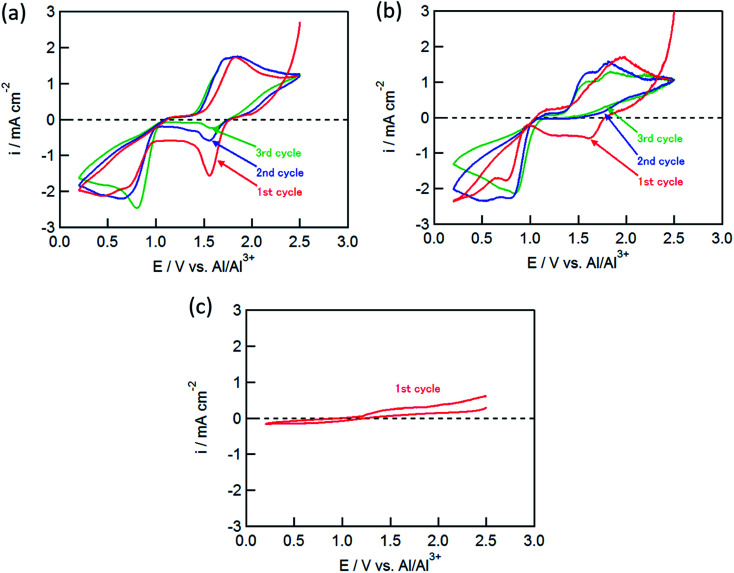
CVs of (a) CuCl_2_, (b) CuCl, and (c) FeCl_2_ electrodes in AlCl_3_/DPSO_2_/toluene solution. Sweep rate, 0.1 mV s^−1^.

Fig. 1 shows the cyclic voltammograms (CVs) for the CuCl_2_, CuCl, and FeCl_2_ pellet electrodes in the AlCl_3_/DPSO_2_/toluene (1 : 10 : 5) solution. As shown in Fig. 1(a), the CuCl_2_ electrode exhibited several redox peaks: cathodic peaks at around 0.6 and 1.6 V, and an anodic peak at 1.8 V. The cathodic peaks are attributable to the Cu^2+^/Cu^+^ and Cu^+^/Cu couples at the higher and lower potentials, respectively.^11^

Similar to eqn (1), the conversion reactions for the CuCl_2_ electrode are

2 Al^3+^ + 3e^−^ + 3Cu(ii)Cl_2_ ⇄ 3Cu(i)Cl + AlCl_3_

3 Al^3+^ + 3e^−^ + 3Cu(i)Cl ⇄ 3Cu(0) + AlCl_3_

The cathodic peak at 1.6 V gradually decreased with an increase in the number of cycles, whereas the cathodic peak around 0.6 V and the anodic peak at 1.8 V did not change, suggesting that the latter two peaks corresponded to the redox couple for Cu^+^/Cu, and that the anodic reaction of Cu^+^ to Cu^2+^ was not observed in this potential range. The CuCl electrode showed similar CVs to the CuCl_2_ electrode as shown in Fig. 1(b); however, the cathodic peak at 1.6 V was not observed in the second cycle, although the electrochemical reduction (eqn (2)) of the partially oxidized CuCl occurred to a small extent in the first cycle. In contrast, electrochemical reactions were not observed for the FeCl_2_ electrode (Fig. 1(c)), which was different from the results of Donahue *et al.* They used reticulated vitreous carbon (RVC) as the current collector; however, this proved deleterious because AlCl_4_^−^ was inserted into carbon during the oxidation.^21^ The charge/discharge curve of the FeCl_3_ positive electrode largely agreed with that for a carbon positive electrode as reported by Dai and co-workers,^21^ suggesting that the positive electrode material was not FeCl_3_ but carbon itself. In this study, the Mo current collector was used as an alternative to the RVC current collector, so the insertion of AlCl_4_^−^ did not occur in the present experiment, clearly indicating that carbon served as the positive electrode material and not FeCl_2_.

**Fig. 2:**
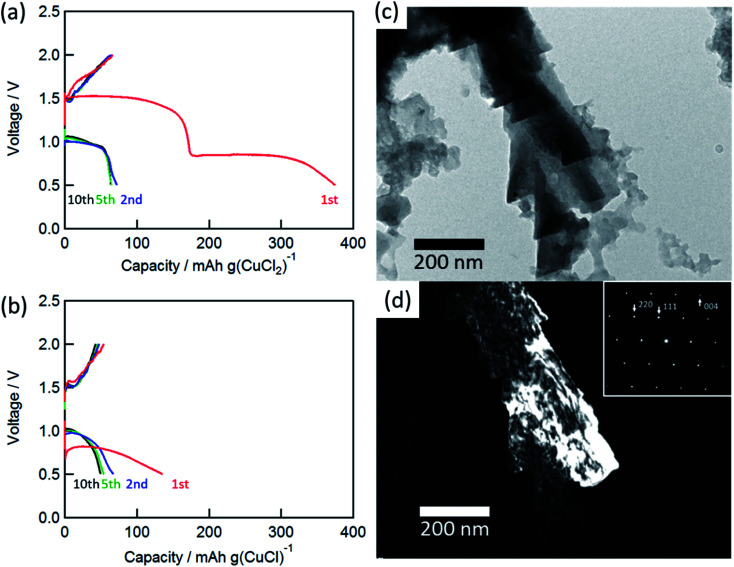
Charge/discharge curves for the rechargeable Al battery with (a) CuCl_2_ and (b) CuCl electrode at a C-rate of 0.028 (11 mA (g-CuCl_2_)^−1^) and 0.043 (17 mA (g-CuCl)^−1^). TEM images: (c) bright-field image and (d) dark-field image of CuCl (111). Inset shows the SAED pattern of the charged electrode.

Fig. 2(a) and (b) show the charge/discharge curves of the CuCl_2_ and CuCl pellet positive electrodes. The initial discharge capacity for the CuCl_2_ electrode was about 370 mA h (g-CuCl_2_)^−1^, which was almost the same as the theoretical capacity (399 mA h (g-CuCl_2_)^−1^). The initial discharge curves had potential plateaus at 0.8 and 1.5 V, and their capacities were similar to each other. Therefore, the two-step anodic reactions (eqn (2) and (3)) proceeded with an initial discharging at the CuCl_2_ electrode. The discharge curve of the CuCl electrode showed only one plateau at 0.8 V, which can be attributed to eqn (3). The energy density was calculated as 425.5 mW h (g-CuCl_2_)^−1^ by multiplying the discharge voltage and discharge capacity. SnS_2_ positive electrode shows the discharge capacity as 392 mA h g^−1^; however, its discharge voltage was below 0.8 V and its energy density was 313.6 mW h g^−1^.^20^ Other conversion type positive electrode materials show lower capacity and almost the same discharge voltage than SnS_2_.^17–19^ Therefore our aluminum battery with CuCl_2_ electrode has the highest energy density comparing with other conversion type electrodes.

The charge and discharge capacities after initial discharging drastically decreased to below 70 mA h (g-CuCl_2_)^−1^, which could be attributed to the electro-elution of copper ions into the electrolyte solution. After the first discharge, the electrolyte solution was taken from the cell and dried, and then elemental analysis of the residue was performed by EDX. The elemental results were as follows: Al 54.22 at%, S 10.28 at%, Cl 33.60 at%, and Cu 1.90 at%, suggesting that a part of active material was dissolved into the solution. Fang *et al.* suggested the modified separator was effective to increase cyclability, and we considered the same type of separator may also be valid for the rechargeable aluminum battery with CuCl_2_ electrode.^22^ To understand the charge/discharge mechanisms, transmission electron microscopy (TEM) measurements were performed.

Fig. 2(c) and (d) show the bright-field and dark-field images for CuCl (111) and the selected-area electron diffraction (SAED) pattern of the charged electrode. The SAED pattern suggested that the discharged active material was CuCl, and the CuCl crystal exhibited a complicated shape on the carbon substrate. Unfortunately, a clear TEM image for the discharged material was not obtained. This result suggested that the active material was converted to CuCl during the charge/discharge cycles.

To understand the charge/discharge mechanism better, the standard electrode potential for each redox reaction was calculated. The two oxidation reactions at the positive electrode and the Al electrodeposition reaction at the negative electrode are represented as follows:

Positive electrode:

4 Cu + Cl^−^ → CuCl + e^−^ *E*° = 0.108 V *vs.* SHE

5 CuCl + Cl^−^ → CuCl_2_ + e^−^ *E*° = 0.936 V *vs.* SHE

Negative electrode:

6 Al^3+^ + 3e^−^ → Al *E*° = −1.68 V *vs.* SHE

The theoretical decomposition voltage 
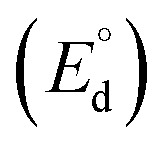
 during the charging process was calculated as 1.78 V by eqn (4) and 2.61 V by eqn (5) from the difference in *E*° values between each positive electrode reaction and the negative electrode reaction in eqn (6). These voltages were higher than the discharge plateau voltages of the CuCl_2_ and CuCl electrodes. This means that the discharge voltage was decreased by overvoltage and the production of CuCl_2_ can occur at charge voltages higher than 2.61 V. Thus, we raised the upper limit of the cut-off voltage to improve the cycle performance of the CuCl_2_ pellet electrode.

**Fig. 3:**
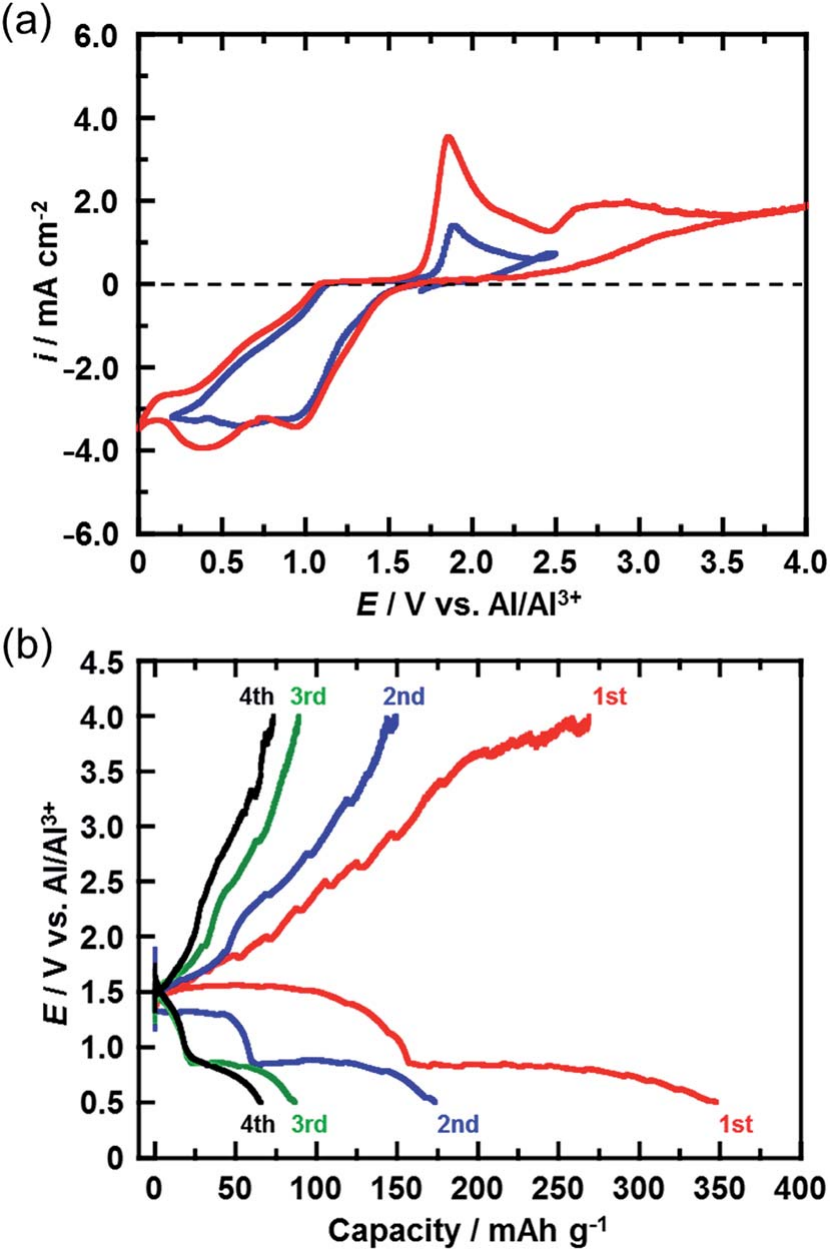
(a) CVs (third cycle) of the CuCl_2_ electrode in AlCl_3_/DPSO_2_/toluene solution for potential sweep ranges of 0–2.5 V and 0–4 V. Sweep rate, 0.1 mV s^−1^. (b) Charge/discharge curves for the rechargeable Al battery with the CuCl_2_ electrode at a C-rate of 0.028 (11 mA (g-CuCl_2_)^−1^). The cut-off voltage during charging was 4 V.

Fig. 3(a) shows the CVs (third cycle) of the CuCl_2_ pellet electrode over a wide potential range (0–4 V) and a narrow potential range (0–2.5 V). In the CV with the narrow potential range, an oxidation peak at about 1.8 V and a broad reduction peak around 0.5–1.0 V were observed. In the CVs with the wide potential range, an additional broad oxidation peak was observed at 2.7 V, suggesting that an upper limit of over 2.7 V is required in order to charge the CuCl_2_ positive electrode, probably according to eqn (5). This charge voltage agreed well with the calculated value of 2.61 V. Fig. 3(b) shows the charge/discharge curves of the CuCl_2_ pellet electrode between 0.5 and 4.0 V. The discharge capacities in the second and third cycles were 175 and 90 mA h (g-CuCl_2_)^−1^, respectively, which were higher than those in the second and third cycles, as the upper limit of the charge potential was 2.0 V (Fig. 2(a)). These findings suggest that charging the CuCl_2_ pellet electrode to the higher voltage was effective, but that the cyclability was insufficient.

**Fig. 4:**
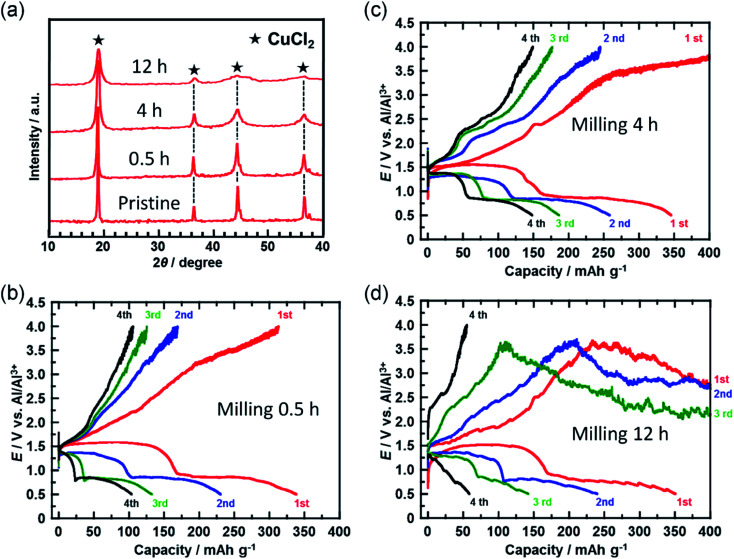
(a) XRD patterns for CuCl_2_ powders ball-milled for different time periods. (b–d) Charge/discharge curves for the rechargeable Al batteries with ball-milled CuCl_2_ electrodes at a C-rate of 0.028 (11 mA (g-CuCl_2_)^−1^). Ball-milling times were (b) 0.5, (c) 4, and (d) 12 h.

In a further attempt to improve cyclability, the size of the CuCl_2_ particles in the prepared electrode was reduced. Fig. 4(a) shows the XRD spectra of the CuCl_2_ powders ball-milled for different time periods. The diffraction peak broadened as the milling time increased, suggesting that the size of the CuCl_2_ particles was reduced by ball-milling. CuCl_2_ powders ball-milled for 0.5, 4, and 12 h are referred to as CuCl_2_[m0.5], CuCl_2_[m4] and CuCl_2_[m12], respectively.

The crystalline size of CuCl_2_ was calculated with the Scherrer equation;7
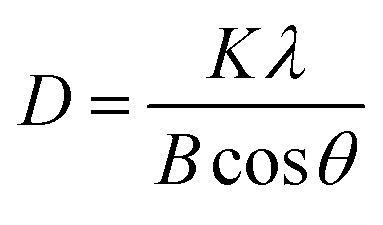
where *D* is the crystalline size (nm), *K* is shape factor (0.9), *λ* is the wavelength of the X-ray, *B* is the full width at half maximum, *θ* is the Bragg angle. Each crystalline size was calculated as 24.0 nm (pristine), 18.0 nm (CuCl_2_[m0.5]), 14.4 nm (CuCl_2_[m4]), and 8.0 nm (CuCl_2_[m12]), respectively.

Fig. 4(b–d) show the charge/discharge curves of the CuCl_2_[m0.5], CuCl_2_[m4], and CuCl_2_[m12] pellet electrodes, respectively. The CuCl_2_[m0.5] and CuCl_2_[m4] electrodes showed better cyclability than the pristine CuCl_2_ electrode, and the CuCl_2_[m4] electrode performed the best of the three electrodes. The discharge capacity at the fifth cycle for the rechargeable Al battery with the CuCl_2_[m4] electrode was 150 mA h (g-CuCl_2_)^−1^, which was about threefold that for the pristine CuCl_2_ electrode. For the CuCl_2_[m12] electrode, the charge process was not terminated. As expected, CuCl_2_[m12] had the smallest particle size as evidenced by having the broadest diffraction peaks. The smaller the particle size, the larger the interfacial surface area for the conversion reaction, thus leading to better electrochemical reversibility. The charging voltage of the CuCl_2_[m12] electrode initially increased to 3.5 V, and then decreased to 3.0 V, suggesting that unfavorable processes occurred. The discharge capacity at the second cycle was 250 mA h (g-CuCl_2_)^−1^, indicating that the positive electrode was not seriously damaged. Decreasing the size of the CuCl_2_ particles increased the surface area of the active material of the positive electrode and may have accelerated the electrochemical decomposition of the electrolyte prior to the charge reactions of eqn (4) and (5), hence the cell voltage did not reach 4.0 V. Based on these results, the rapid reduction of the discharge capacity of the CuCl_2_ electrode, as depicted in Fig. 2(b), might be attributable to the low cut-off voltage and the large particle size of the CuCl_2_ particles.

## 4. Conclusion

Three transition metal chloride salts were examined as potential candidates for the conversion-type positive electrode active materials for rechargeable Al batteries. The rechargeable Al battery with the CuCl_2_ and CuCl positive electrodes worked well. For the CuCl_2_ electrode, plateaus in electrode potential were observed around 0.8 and 1.5 V during the discharge process. The initial discharge capacity of the CuCl_2_ electrode was similar to its theoretical value of 370 mA h (g-CuCl_2_)^−1^ and energy density was 392 mA h g^−1^. This value is higher than that of other conversion-type positive electrode materials for the rechargeable Al battery. However, the discharge capacity at the second cycle rapidly decreased to less than 20% of the theoretical value. To improve the cyclability of the CuCl_2_ positive electrode, the upper cut-off voltage was increased to 4.0 V and the size of the CuCl_2_ particles was decreased by mechanical milling. Consequently, the discharge capacity at the fifth cycle for the electrode made with the CuCl_2_ powder that was ball-milled for 4 h was 150 mA h (g-CuCl_2_)^−1^, which is about threefold that for the pristine CuCl_2_ electrode.

## Conflicts of interest

There are no conflicts to declare.

## Supplementary Material
